# Effects of Chondroitinase ABC-Mediated Proteoglycan Digestion on Decellularization and Recellularization of Articular Cartilage

**DOI:** 10.1371/journal.pone.0158976

**Published:** 2016-07-08

**Authors:** Catherine A. Bautista, Hee Jun Park, Courtney M. Mazur, Roy K. Aaron, Bahar Bilgen

**Affiliations:** 1 Center for Biomedical Engineering, Brown University, Providence, Rhode Island, United States of America; 2 Division of Biology and Medicine, Brown University, Providence, Rhode Island, United States of America; 3 Department of Orthopaedics, Warren Alpert Brown Medical School of Brown University/Rhode Island Hospital, Providence, Rhode Island, United States of America; 4 Providence VA Medical Center, Providence, Rhode Island, United States of America; Michigan Technological University, UNITED STATES

## Abstract

Articular cartilage has a limited capacity to heal itself and thus focal defects often result in the development of osteoarthritis. Current cartilage tissue engineering strategies seek to regenerate injured tissue by creating scaffolds that aim to mimic the unique structure and composition of native articular cartilage. Decellularization is a novel strategy that aims to preserve the bioactive factors and 3D biophysical environment of the native extracellular matrix while removing potentially immunogenic factors. The purpose of this study was to develop a procedure that can enable decellularization and recellularization of intact articular cartilage matrix. Full-thickness porcine articular cartilage plugs were decellularized with a series of freeze-thaw cycles and 0.1% (w/v) sodium dodecyl sulfate detergent cycles. Chondroitinase ABC (ChABC) was applied before the detergent cycles to digest glycosaminoglycans in order to enhance donor chondrocyte removal and seeded cell migration. Porcine synovium-derived mesenchymal stem cells were seeded onto the decellularized cartilage scaffolds and cultured for up to 28 days. The optimized decellularization protocol removed 94% of native DNA per sample wet weight, while collagen content and alignment were preserved. Glycosaminoglycan depletion prior to the detergent cycles increased removal of nuclear material. Seeded cells infiltrated up to 100 μm into the cartilage deep zone after 28 days in culture. ChABC treatment enhances decellularization of the relatively dense, impermeable articular cartilage by reducing glycosaminoglycan content. ChABC treatment did not appear to affect cell migration during recellularization under static, *in vitro* culture, highlighting the need for more dynamic seeding methods.

## Introduction

Articular cartilage defects result in joint pain and often develop into osteoarthritis over time [1]. Osteoarthritis is the most common joint disease in the US, affecting an estimated 27 million Americans [2]. Current clinical therapies such as abrasion arthroplasty and microfracture for articular cartilage defects are insufficient to restore the functionality of the load-bearing cartilage [3]. Implantation of tissue-engineered cartilage has not been successful in the past decades, either [4–7]. The major roadblock has been the inability to mimic the unique native articular cartilage ultrastructure that provides the ability to withstand compressive and shear forces within the joint. The lack of functional mechanical strength and physiological ultrastructure in engineered cartilage has resulted in the breakdown of neocartilage *in vivo* and the failure of cartilage replacement strategies in joints. Implantation of osteochondral allografts could be an alternative; however, immune rejection, availability of fresh tissue, and storage conditions limit feasibility [8]. Osteochondral autograft transplantation allows higher patient post-surgery activity levels than microfracture surgery because it preserves the hyaline cartilage composition and structure, including the collagen II ultrastructure [9, 10]. However, autograft transplantation is limited in its availability and results in donor site morbidity. To overcome these limitations, several groups have used devitalized cartilage and bone fragments as raw materials without an intact ultrastructure to reconstitute osteochondral matrices [11–22]. While this method provides versatile bioactive building blocks for engineering cartilage, disruption of the matrix structural integrity eliminates its strength at the tissue-level and much of its intrinsic biophysical cues at the cellular level.

The articular cartilage extracellular matrix (ECM) has a highly specialized architecture that is zonally organized: the superficial zone consists mostly of collagen II fibers aligned parallel to the articular surface to resist shear forces, whereas the deep zone consists of the same fibers aligned perpendicularly to the bone interface to absorb compressive loads [23]. Water and proteoglycans occupy the remaining space and maintain hydrostatic pressure, which provides much of the tissue’s compressive strength. In addition to defining the tissue’s mechanical properties, the physical and biochemical properties of the articular cartilage ECM plays a major role in maintaining the chondrogenic phenotype [24–26].

Decellularization of intact cartilage matrix would provide an ideal scaffold for articular cartilage tissue engineering by preserving the unique ultrastructure and bioactivity of the native tissue while removing immunogenic factors [11, 27–29]. With reduced antigen content, decellularized allogeneic and xenogeneic donor tissue would reduce the need for fresh allografts and autografts [30]. Storage of an acellular scaffold would have a reduced risk of protease-mediated ECM degradation and thus lengthen shelf-life relative to that of fresh autografts and allografts [31].

Decellularization has been extensively explored in a wide variety of tissues and a number of native ECM-based scaffolds have successfully been applied at the clinical level [32]. Several groups have decellularized intact articular cartilage using freeze-thaw cycles, detergents and enzymatic removal of DNA [33–36]. Others have used chemical decellularization methods on nasal septal cartilage, however recellularization attempts have resulted in inhomogeneous cell seeding [37]. Recellularization of cartilage remains a challenge, likely due to the relatively dense proteoglycan and collagen matrix.

In this study, we hypothesized that specific reduction of proteogylcans in the cartilage matrix will enhance decellularization without compromising the collagen network. We employed the enzyme chondroitinase ABC (chABC) to digest aggrecan, the largest and most abundant proteoglycan in articular cartilage, in combination with a freeze-thaw and detergent-based decellularization protocol on porcine articular cartilage [35]. We characterized the decellularized cartilage scaffolds through mechanical, biochemical and histological assessments. In addition, we seeded synovium-derived mesenchymal stem cells on the decellularized cartilage scaffolds to investigate *in vitro* recellularization. This approach resulted in enhanced removal of nuclear material without destruction of collagen content or alignment, which could provide mechanical support and bioactive factors in an *in vivo* setting.

## Materials and Methods

### Cell and Tissue Harvest

Postmortem tissue harvest from pig cadavers was carried out in accordance with the Lifespan Institutional Animal Care and Use Committee (IACUC) Policy for the Responsible Conduct of Animal Research and Use of Central Research Facilities, which does not require review of postmortem tissue harvesting from cadavers. Full-thickness articular cartilage was harvested from the distal femurs of a 3-month-old female Yorkshire pig, after euthanization was carried out by another IACUC approved protocol at Rhode Island Hospital. Synovial membrane was harvested from the tibiofemoral joints from a 4-month-old female pig legs purchased from a local abattoir (RI Beef and Veal, Johnston, RI). 4-mm-diameter cylindrical cartilage plugs of heights ranging from 1 mm to 4 mm were excised using biopsy punches. Each plug was weighed then stored at -20°C in a 1.8-ml cryotube with a strip of PBS-moistened filter paper. Plugs were grouped such that there were no significant differences among average group weights. Synovium-derived mesenchymal stem cells (SDSCs) were isolated from the synovial membranes according to previously developed protocols [38].

### Decellularization

**Fig 1:**
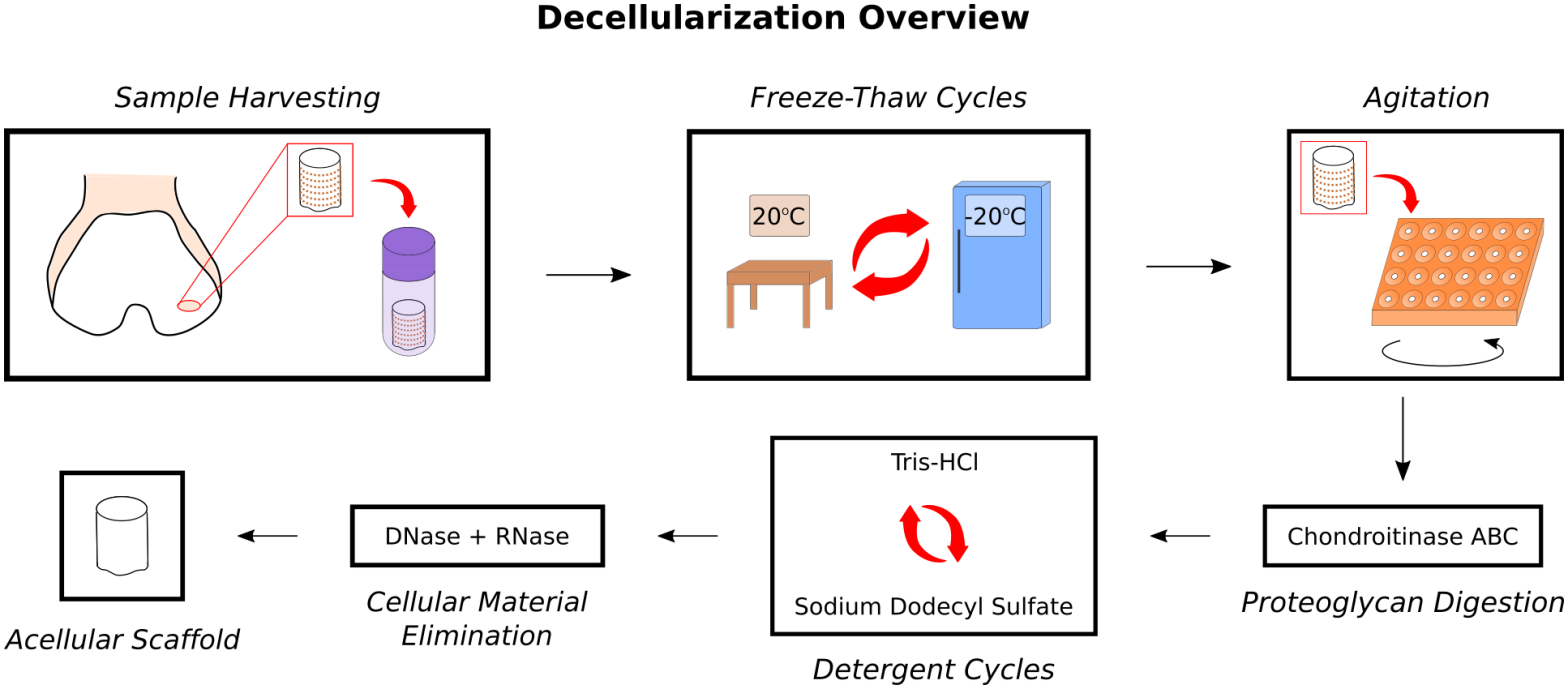
Overview of optimized decellularization procedure used in study. Harvested cartilage plugs were frozen at -20°C then subjected to two dry FTCs followed by two wet FTCs with hypotonic buffer. Plugs were then incubated in multiwell plates on an orbital shaker with high frequency agitation (220 RPM) for the remaining steps. Samples were submerged in a 0.125 U/ml chABC solution to digest GAGs. This was followed by three wash cycles of alternating hypotonic buffer and 0.1% (w/v) SDS detergent to increase cell membrane permeability and promote cell lysis. Finally, DNase and RNase were added to degrade nuclear material.

The decellularization procedures employed in this study were based on a protocol developed by Kheir et al. [35] and optimized in our lab [39, 40]. Plugs underwent two “dry” freeze-thaw cycles (FTCs), followed by two “wet” FTCs. One dry FTC consisted of being thawed completely at room temperature (RT) and being returned to the -20°C freezer overnight. For each wet FTC, the plugs were allowed to thaw at RT, fresh hypotonic buffer (10 mM tris-HCl) was added to each tube, and then the plugs were frozen overnight at -20°C. For the remaining steps, the plugs were transferred to 48-well flat-bottom plates and agitated at 220 RPM on an orbital shaker. ChABC treatment was added to the decellularization procedure to maximize glycosaminoglycans (GAG) extraction; chABC application was based on the protocol developed by Schmidt et al. [41] and optimized for decellularization in previous experiments in our laboratory [40]. Each plug was incubated in 0.125 U/ml chABC from *Proteus vulgaris* (Sigma-Aldrich) at 37°C for 24 h; phosphate-buffered saline (PBS) was used as a control. The plugs then underwent three anionic detergent cycles, each consisting of two steps. The first step was incubation in hypotonic buffer at 45°C for 24 h. The second step was incubation in 0.1% (w/v) sodium dodecyl sulfate (SDS) detergent at 45°C for 24 h. After three detergent cycles, the plugs were rinsed four times in PBS with 2.95 μl/ml Protease Inhibitor Cocktail (PIC; Sigma-Aldrich). The plugs were then treated with nucleases (100 U/ml DNase, 1 U/ml RNase). Plugs were rinsed in PBS with PIC, then decontaminated in 0.1% (v/v) peracetic acid. Finally, samples were rinsed three times in PBS. Fig 1 summarizes the optimized decellularization procedure.

### Recellularization

**Fig 2:**
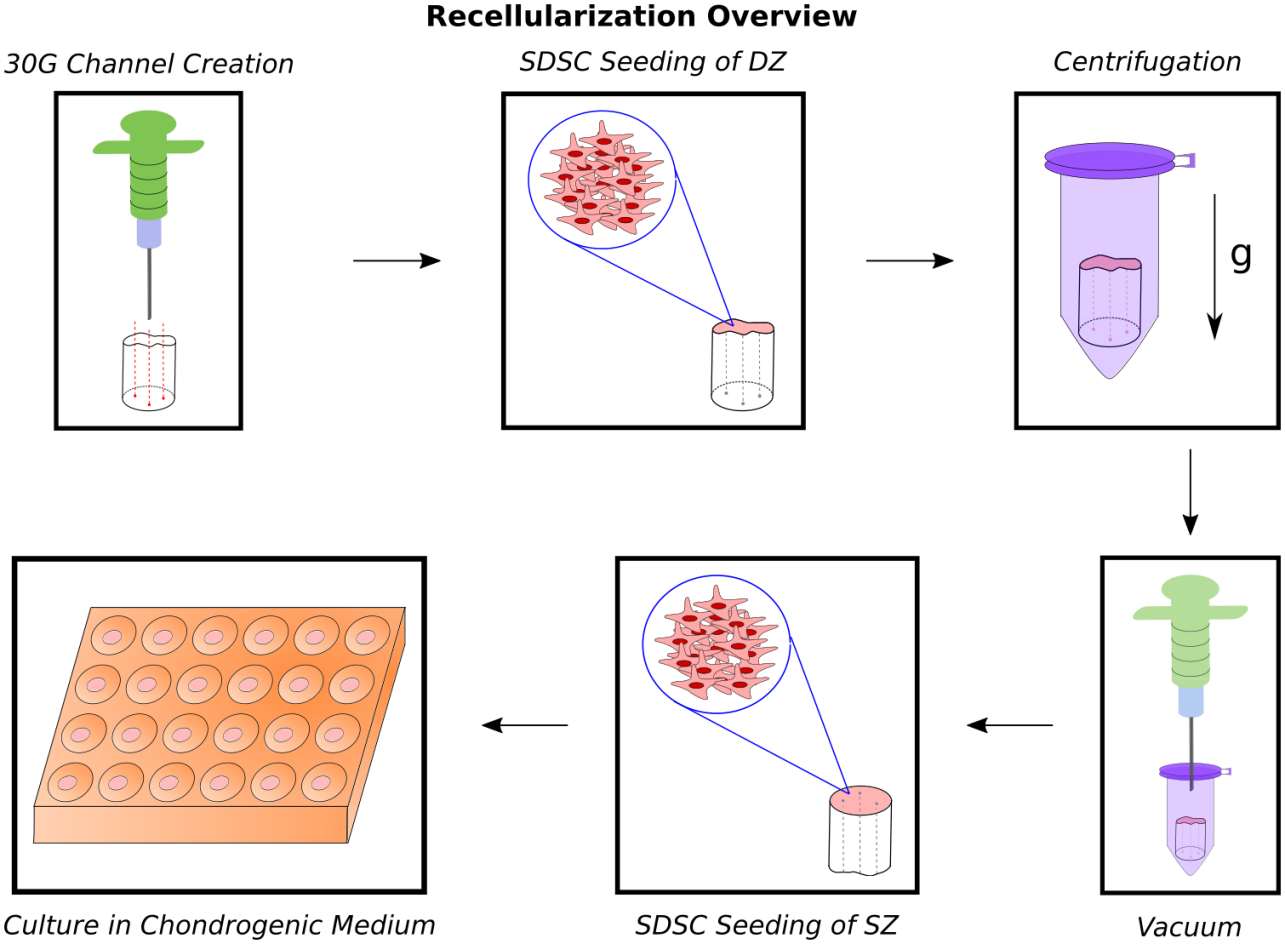
Overview of recellularization procedure. Three channels were created in the decellularized cartilage scaffolds. The cells were pipetted onto the scaffolds in two steps. For the first step, half of the cell suspension was pipetted onto the deep zone (DZ) surface and the samples were centrifuged. A syringe was used to create a vacuum in the centrifuge tubes to maximize seeding. For the second step, the remaining cell suspension was pipetted onto the superficial zone (SZ) surface. Samples were cultured in serum-free chondrogenic medium.

Passage 2 SDSCs were labeled with Vybrant^®^ DiI Cell Labeling Solution (Life Technologies) prior to seeding. Fig 2 summarizes the recellularization procedure. The acellular cartilage scaffolds were transferred to 24-well plates and immersed in 10% fetal bovine serum-supplemented culture medium for 24 h before seeding. A 30-gauge (310 μm-diameter) needle was used to create three channels in each scaffold to enhance cellular infiltration throughout the depth of the plug. The cells were seeded onto the scaffolds in two steps for a total density of 30 million SDSCs per cc sample. For the first seeding step, scaffolds were transferred to individual 1.5-ml centrifuge tubes and half of the seeding volume was pipetted onto the deep zone surface. The tubes were then centrifuged at 400g for 5 min to force the cell suspension into the matrix [42]. A 10-ml syringe was used to create a vacuum in each tube for 10 s in order to maximize cell seeding [43]. After 1 h scaffolds were transferred to 24-well plates. For the second seeding step, the remaining cell suspension was pipetted onto the superficial zone surface. After 1 h, 2 ml serum-free medium [Dulbecco’s Modified Eagle Medium with 25 mM D-glucose and 1 mM sodium pyruvate (Gibco), 1% ITS+ Premix (Corning), 1% Penicillin-Streptomycin-Glutamine (Gibco), 0.4% Amphotericin B (Gibco), 1% Nonessential Amino Acids (Sigma), 0.4 mM L-proline] was added to each well and the plates were placed on an orbital shaker at 50 RPM at 37°C and 5% CO_2_ overnight. The next day, the plates were removed from the orbital shaker and chondrogenic factors [50 μg/ml ascorbate-2-phosphate, 100 nM dexamethasone, 10 ng/ml TGF-*β*1 (R&D Systems)] were added to the medium. 50% of the media was replaced every 2-3 days. Seeded scaffolds were cultured for 6, 14, or 28 days.

### Biochemical Analyses

Samples were lyophilized, weighed, and digested overnight at 60°C in 0.125 U/ml papain from papaya latex (Sigma-Aldrich). GAG, double-stranded DNA (dsDNA), and collagen contents were measured using the 1,9-dimethylmethylene blue (DMMB) assay, the PicoGreen^®^ dsDNA Quantitation Assay (Life Technologies), and the hydroxyproline assay (Sigma-Aldrich), respectively. For the hydroxyproline assay, we used a hydroxyproline:collagen ratio of 1:7.64 [44]. Biochemical content was normalized by pre-decellularization sample wet weight (WW) or post-decellularization sample dry weight (DW).

### Histology

Samples (*n* = 3) were fixed in 4% neutral buffered formalin, embedded, and cryosectioned. Sections were stained with hematoxylin and eosin (H&E) for nuclei or Safranin-O and Fast Green for proteoglycans, then imaged using bright-field microscopy at 100X. Sections were also stained with Picrosirius Red and imaged with cross-polarized light microscopy for collagen fiber alignment. To observe cellular infiltration, recellularized sections were labeled with 4’,6-diamidino-2-phenylindole (DAPI), then imaged with differential interference contrast and fluorescence microscopy at 100X or 200X.

### Mechanical Testing

Mechanical testing was performed in unconfined compression using Instron Electropuls E1000. First, a tare load of 0.02 N was applied to the superficial zone surface of the plug for 5 min. Subsequently, a stress relaxation test was performed at 10% strain until equilibrium was reached. The equilibrium modulus was calculated using the stress at the end of the relaxation period.

### Statistical Analyses

One-way analysis of variance with Tukey’s post hoc test (*p*<0.05) was used to assess the differences in biochemical and mechanical properties [45]. Numerical data are represented as arithmetic mean of n = 5–13 samples ± standard error of the mean (SEM).

## Results

### Decellularization

**Fig 3:**
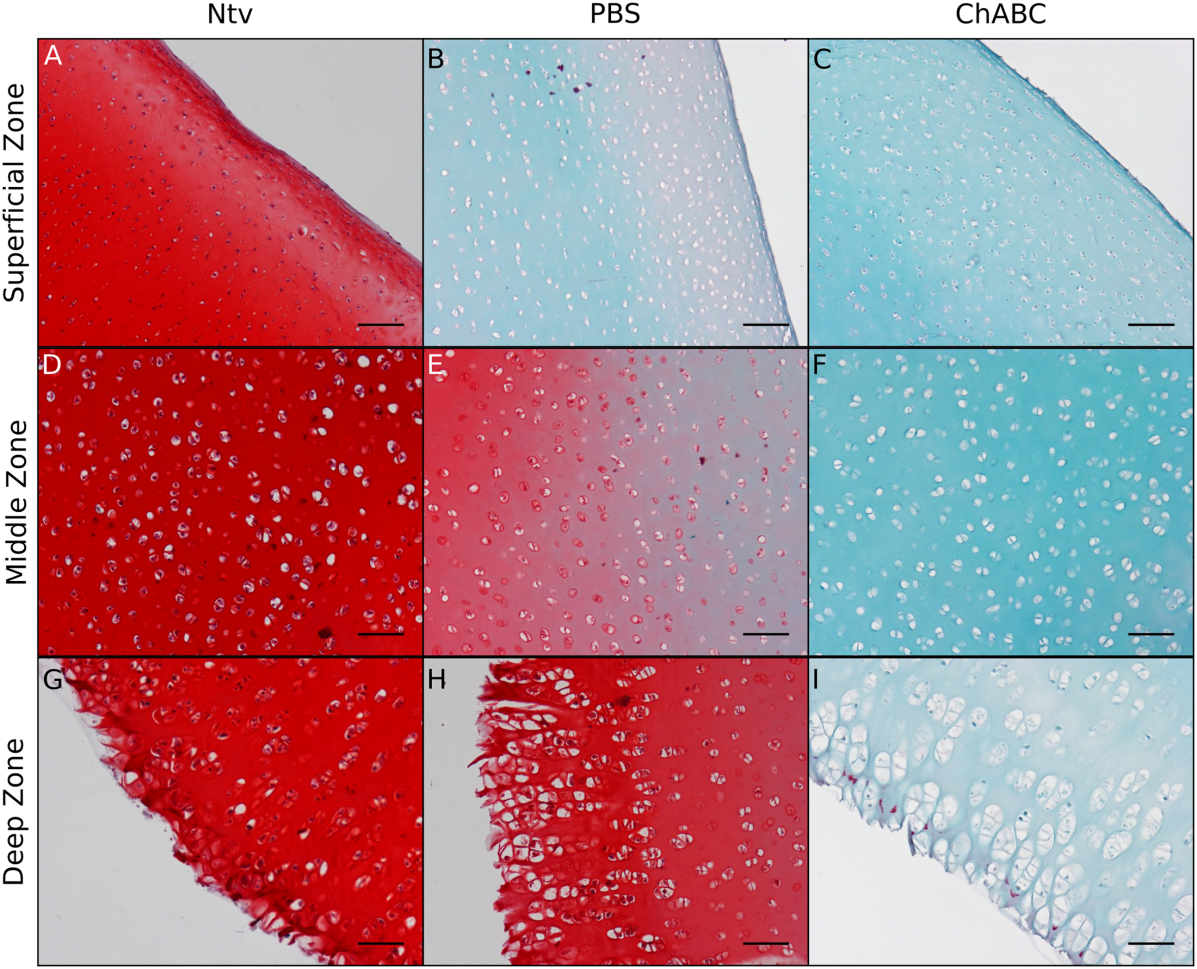
Safranin-O and Fast Green staining of longitudinal cross sections of decellularized cartilage. Compared to native cartilage (Ntv), both groups of decellularized cartilage had significantly less proteoglycan staining, with more proteoglycan removal in the chABC-treated scaffolds compared to the PBS controls. Scale bar = 100 μm and magnification = 100X.

**Fig 4:**
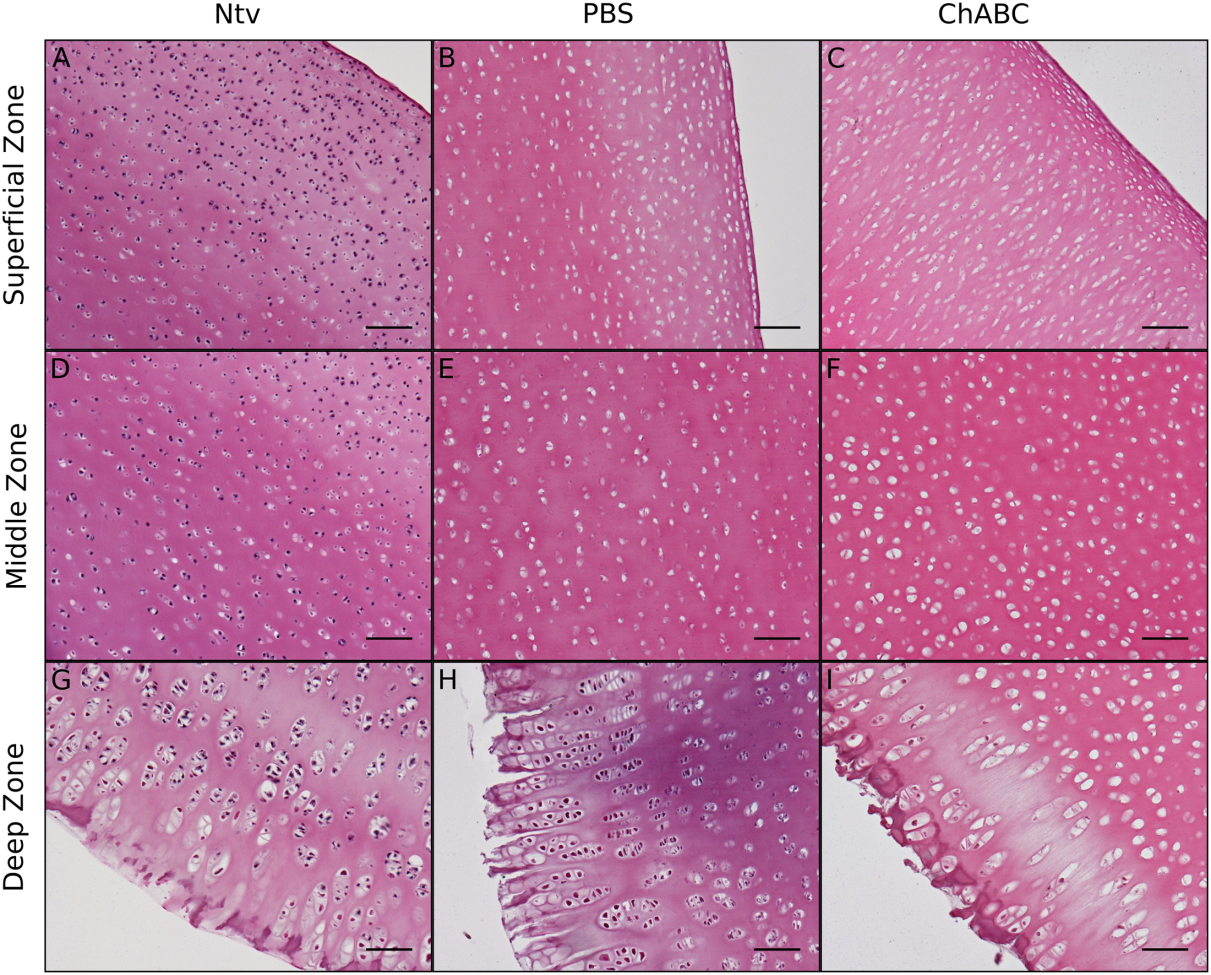
H&E staining of longitudinal cross sections of decellularized cartilage. Compared to native cartilage (Ntv), both groups of decellularized cartilage contained less cellular material in the superficial, middle, and deep zones. The PBS group had slightly more residual nucleic material than the chABC group, especially in the deep zone (G-I). Scale bar = 100 μm and magnification = 100X.

Safranin-O and Fast Green staining of decellularized cartilage showed a trend of decreasing proteoglycan content with the addition of chABC (Fig 3). H&E staining of decellularized cartilage showed removal of cellular material in lacunae in both decellularized groups (Fig 4). Dark nucleic acid staining was virtually nonexistent in the superficial and middle zones of the PBS- and chABC-treated scaffolds, indicating that the decellularization procedure successfully removed most nucleic material from the cartilage in those regions (Fig 4A–4F). There appeared to be more cellular remains in the deep zone of the PBS group (Fig 4H), where there were also more proteoglycans (Fig 3H), compared to the same region in the chABC group (Figs 4I & 3I).

**Fig 5:**
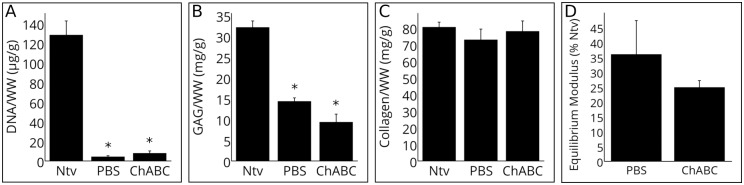
Biochemical content and mechanical properties of decellularized cartilage. (A) dsDNA/WW, as quantified by the PicoGreen assay. The decellularization procedure significantly reduced dsDNA/WW from native cartilage (Ntv), with no significant difference between the PBS and chABC groups. (B) GAG/WW, as quantified by the DMMB assay. Both decellularized groups had significantly lower GAG compared to native cartilage. (C) Collagen/WW, as quantified by the hydroxyproline assay. Collagen content was maintained after both decellularization procedures. (D) Equilibrium modulus, represented as percent of pre-decellularization equilibrium modulus. Both groups showed a decrease in equilibrium modulus after decellularization. Data is plotted as arithmetic mean ± SEM; asterisks denote significant differences from native cartilage (*p*<0.05; *n* = 5–13).

Native dsDNA/WW was reduced by over 90% in both PBS and chABC scaffolds (Fig 5A); *p*<0.05), supporting the results of the H&E staining. dsDNA/DW was 503.7, 34.6 and 47.6 ng/mg for the native, PBS, and chABC groups, respectively. The dsDNA/WW of the PBS and chABC groups were not significantly different. The DMMB assay results confirmed that more GAG/WW was removed in the chABC group than in the PBS group, although this difference was not significant (Fig 5B; *p*>0.05). Both decellularized groups had significantly less GAG/WW compared to native cartilage (*p*<0.05). The hydroxyproline assay showed that collagen content was maintained after both decellularization procedures (Fig 5C; *p*>0.05).

The same subset of plugs were mechanically tested before and after decellularization, so that each specimen’s stiffness could be compared to its original stiffness. Fig 5D shows the equilibrium modulus of the decellularized plugs as a percent of their pre-decellularization equilibrium modulus. Both groups significantly decreased in stiffness after decellularization. ChABC-treated scaffolds had a larger decrease in equilibrium modulus compared with control PBS-treated scaffolds, although this difference was not significant (*p*>0.05). Mean equilibrium modulus of chABC-treated scaffolds decreased from 145 kPa (range from 81 kPa to 200 kPa) to 35 kPa (range from 19 kPa to 56 kPa). Mean equilibrium modulus of PBS-treated scaffolds decreased from 155 kPa (range from 87 kPa to 278 kPa) to 60 kPa (range from 9 kPa to 174 kPa). Table 1 summarizes the biochemical and mechanical properties of both test groups as percentages of native cartilage values.

**Table 1:** Mean Biochemical and Mechanical Properties of Decellularized Cartilage as Percentages of Native Values.

Group	GAG/WW	dsDNA/WW	Collagen/WW	Equilibrium Modulus
PBS	44.5±3.3%	3.2±0.7%	90.5±8.8%	35.9±11.3%
ChABC	29.0±6.1%	6.0±1.8%	96.8±8.7%	24.8±2.2%

Values expressed as % native value ± SEM.

**Fig 6:**
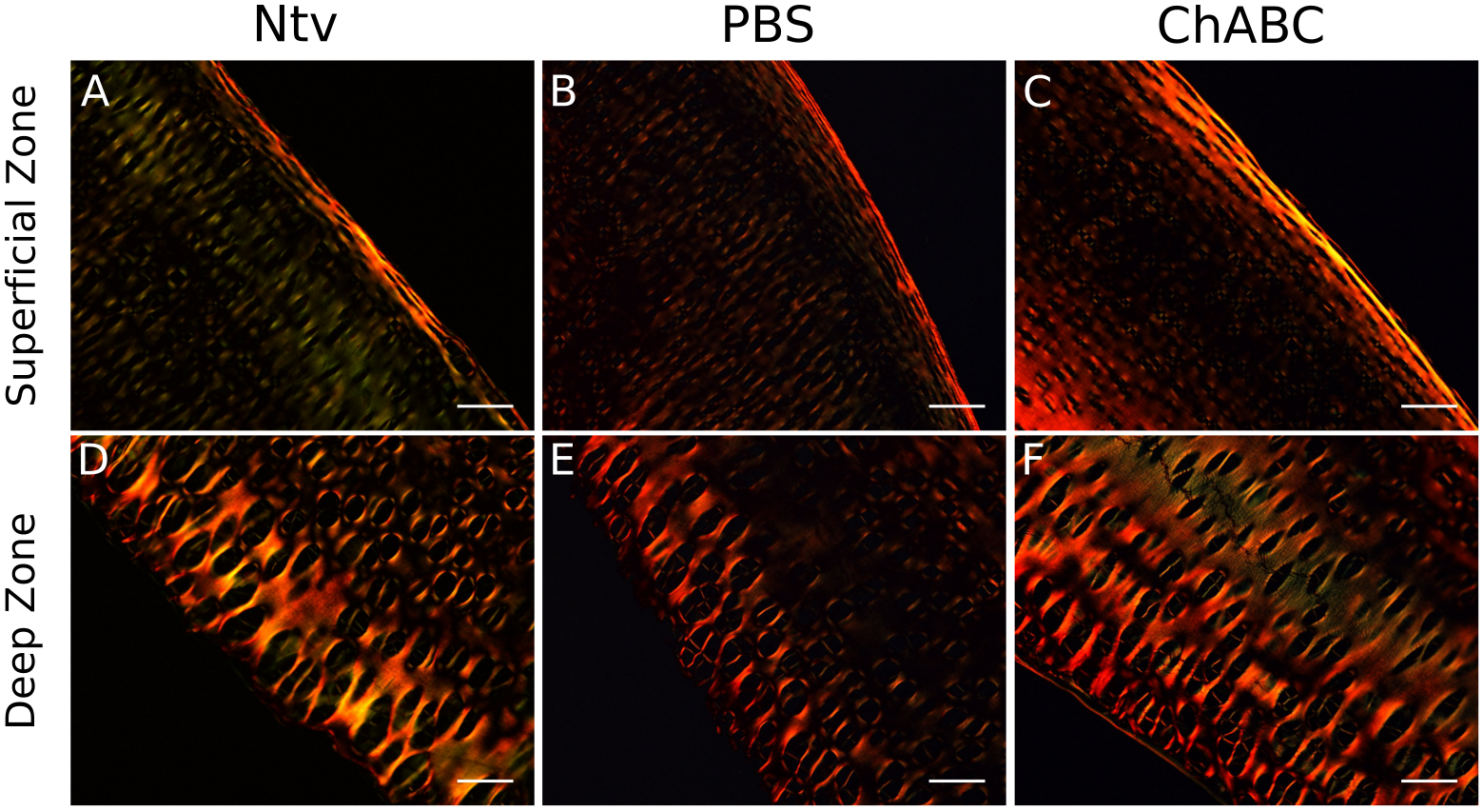
Picrosirius Red staining of longitudinal cross sections of decellularized cartilage, viewed with cross-polarized light. Regions of high collagen fiber alignment appear with greater intensity. Collagen fiber alignment in the superficial (A-C) and deep (D-F) zones was maintained after decellularization. Scale bar = 100 μm and magnification = 100X.

Cross-polarized light imaging of Picrosirius Red-stained sections showed preservation of collagen fiber alignment after decellularization in the superficial zone (Fig 6A–6C) as well as the deep zone (Fig 6D–6F), as indicated by the bright intensity along the edges of the tissue.

### Recellularization

**Fig 7:**
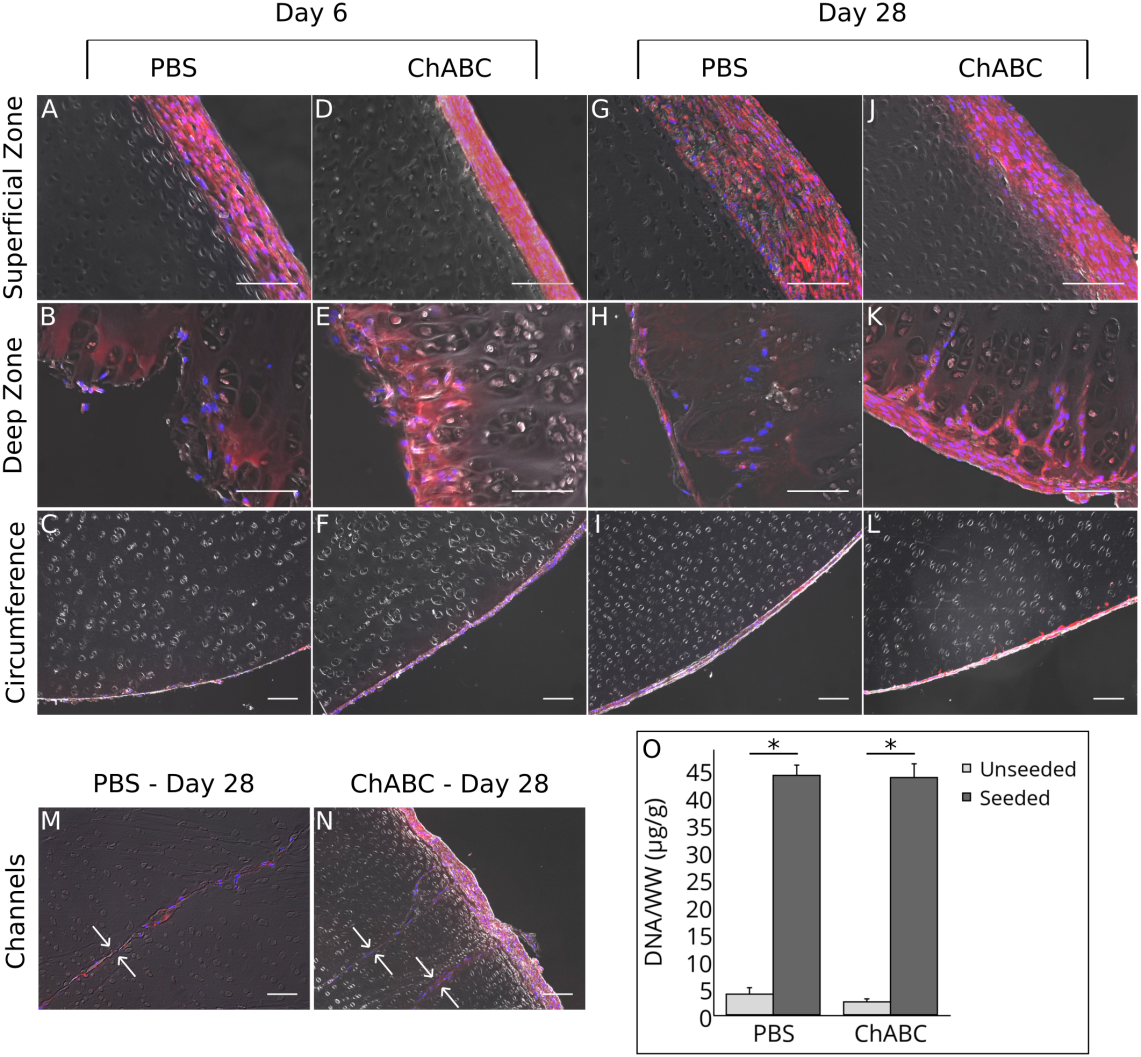
Summary of Recellularization Results. (A-N) Fluorescent labeling of seeded SDSCs. Seeded SDSCs were labeled with red DiI and all cells (seeded and native) were labeled with blue DAPI; fluorescent images were superimposed onto differential interference contrast images to more easily view infiltration. (A-F) Within 6 days, SDSCs attached to all outer surfaces, with some infiltration into the deep zone. (G-L) After 28 days, the outer cell layers grew thicker and there were generally more cells in the deep zone. (M-N) SDSCs were found throughout the lengths of the channels (channel borders indicated by small white arrows). (O) dsDNA/WW after 28 days in culture, as quantified by the PicoGreen assay. The seeded groups had significantly more dsDNA/WW compared to their respective unseeded controls, with no significant difference between the seeded PBS and chABC groups. For histology, scale bar = 100 μm and magnification = 200X for superficial and middle zones and 100X for circumference and channels. For biochemistry, data is plotted as arithmetic mean ± SEM; asterisks denote significant differences between groups (*p*<0.05; *n* = 6–10).

Fluorescent labeling of seeded SDSCs showed adhesion to all outer surfaces of the scaffolds after 6 days in culture (Fig 7A–7F). Infiltration occurred as early as 6 days in culture, but only in the deep zone (Fig 7C & 7F). After 28 days in culture, outer cell layers grew thicker and infiltrating cells migrated up to 100 μm into the deep zone (Fig 7G–7L). Seeded cells were found throughout the channels created by the 30-gauge needle (Fig 7M and 7N), with little evidence of radial infiltration into the surrounding ECM after 28 days. dsDNA/WW after the 28-day culture period was significantly higher in the seeded scaffolds compared to unseeded ones, indicating successful SDSC attachment (Fig 7O; *p*<0.05). There was no significant difference in dsDNA/WW between the seeded PBS and chABC groups. GAG/WW remained constant before and after the 28-day culture period for both chABC and PBS groups (data not shown; *p*>0.05; *n* = 6–10). There was no significant difference in equilibrium modulus before and after the 28-day culture period for both chABC- and PBS-treated scaffolds (data not shown; *p*>0.05; *n* = 5–13).

## Discussion

In this study, we demonstrated that chABC treatment during decellularization enhances removal of cellular material from lacunae compared to the control treatment. We established a protocol for controlled GAG digestion, while preserving the native collagen matrix, by applying chABC to the cartilage during decellularization. Chondroitinase ABC application increases GAG digestion, as suggested by the Safranin-O and Fast Green staining, and increases cellular material removal, as shown by H&E staining. After 28 days of static *in vitro* culture, we demonstrated cell infiltration as far as 100 μm into the deep zone and cell attachment on the outer surfaces of each plug, as shown by fluorescent cell labeling and imaging.

Our decellularization protocol using chABC resulted in a 94% decrease in dsDNA/WW, in keeping with previous studies [34, 35]. Furthermore, similar to these previous studies, the final dsDNA content reported here falls below the 50 ng/mg ECM dry weight threshold for prevention of adverse host reactions described by Crapo et al. [46]. Although there was no significant difference in dsDNA/WW between the PBS-treated and chABC-treated scaffolds, there appeared to be less nucleic material staining with H&E in the chABC-treated plugs, suggesting that chABC treatment enhances removal of cellular remains. While Kheir et al. found no evidence of inflammation after subcutaneous implantation despite evidence of chondrocyte remains in the ECM, F4/80+ macrophage infiltration was observed [35]; such occurences have been associated with both host rejection and constructive remodeling [47]. Therefore, increased removal of donor chondrocytes may be beneficial for preventing a negative immune response upon implantation [48].

The approach of digesting ECM components to improve diffusion of decellularization agents has been employed in other tissues. Utomo et al. found that the addition of elastase treatment to the Kheir et al. protocol [35] resulted in greater removal of residual cellular material in human ear cartilage [49]. While ECM digestion can improve decellularization, it also compromises the mechanical strength of the tissue. GAG depletion has repeatedly been shown to decrease the compressive stiffness of articular cartilage because the loss of the charged molecules no longer supports hydrostatic pressure [41, 50–53]. The decrease in GAG content and compressive stiffness relative to native cartilage observed in this study is comparable to similar losses observed in other cartilage decellularization studies [35, 37, 50]. We hypothesize that, once successful repopulation occurs, the scaffolds will promote relatively rapid synthesis of GAGs to replace those that were lost in decellularization. Several studies have shown that, in tissue-engineered cartilage, chABC treatment depleted GAG and decreased compressive stiffness; however after two weeks of culture, these losses showed marked recovery [54–56]. Therefore, GAG depletion may be considered a temporary consequence.

Our decellularization procedure successfully preserved the collagen content and fiber alignment of native tissue, consistent with the findings of other studies that used SDS detergent to decellularize intact cartilage [35, 36, 49]. We hypothesize that keeping the hyaline cartilage ultrastructure intact will induce chondrogenesis and prevent fibrocartilage formation upon *in vivo* implantation, which is a major problem in the widely utilized microfracture surgery [57].

*In vitro* recellularization of decellularized cartilage is a major challenge for *in vivo* success. Because the high density of the articular cartilage ECM physically inhibits cell migration, we investigated the effects of GAG depletion on recellularization potential and characterized the interactions of seeded cells with scaffolds in *in vitro* culture. Seeded SDSCs attached to all surfaces of the scaffolds, regardless of their GAG content. These outer layers of cells and ECM grew thicker from Day 6 to Day 28 in culture, suggesting that the decellularized cartilage provided a proliferative environment to the SDSCs. A thicker layer of cells formed on the superficial zone. This could be due to the initial incubation period, when the plugs were all placed with superficial zone facing upwards in the 24-well plates. After the first media change, orientation of the plugs in their wells was random, which may account for differences in thicknesses of new cell layers from sample to sample. After 28 days in culture, we observed limited cell infiltration in porcine articular cartilage. Our infiltration results are similar to those of Schwarz et al., who found seeded chondrocytes 100–150 μm deep in decellularized porcine native septal cartilage after 28 days in culture [37]. However, there did not appear to be a difference in either the number of cells infiltrating or the migration distances between the chABC-treated group and the control group, suggesting that removal of GAGs did not meaningfully increase the porosity of the matrix. Utomo et al. cultured bone marrow-derived mesenchymal stem cells with acellular ear cartilage for 21 days with no evidence of infiltration into the scaffold [49]. Higher ECM porosity may be necessary for efficient recellularization under static conditions.

Seeded cells filled the 310-μm-diameter channels, showing cell attachment in the inner regions of the scaffold. A recent study by Juran et al. has shown that, by introducing an array of 120-μm-diameter channels into decellularized temporomandibular joint discs, *in vitro* cellular infiltration into the matrix increased without significantly affecting the compressive modulus [58]. The higher degree of cellular infiltration seen by Juran et al. could be due to the more permeable, softer nature of temporomandibular joint disc cartilage in comparison to distal femur articular cartilage [59, 60]. Luo et al., who used hyaluronidase to eliminate GAGs from decellularized porcine articular cartilage, observed limited (under 100 μm) cell migration from 400-μm-diameter channels after 28 days in culture [33]. In the current study, there was no evidence of radial cell migration from the channels; the addition of rotational culture, as in the Luo et al. study, may promote radial migration by improving seeded cell viability and distribution [50].

Dynamic culture environments usually improve cell seeding efficiency on porous scaffolds over static cultures. The decellularized scaffold has a low porosity and this likely requires a higher degree of dynamic physical stimuli to induce cell infiltration. Combination of these acellular, GAG-depleted scaffolds with bone marrow stimulation *in vivo* may enhance cell migration into the matrix. Wang et al. found no evidence of infiltration 12 weeks after implantation of unseeded decellularized ear cartilage in a leporine distal femur model [61]; however, they did not instigate bone marrow flow through the matrix, as in autologous matrix-induced chondrogenesis surgery [62]. They demonstrated that the decellularized cartilage that was pre-cultured for three weeks with adipose-derived mesenchymal stem cells contained cells throughout the ECM 12 weeks after implantation. However, acellular scaffolds may be more clinically relevant because of their off-the-shelf potential and fewer regulatory obstacles. Our results are significant because decellularized articular cartilage scaffolds are more physiologically relevant for implantation into load-bearing joints. Future studies should focus on developing more dynamic cell seeding methods, possibly involving flow perfusion culture to better mimic bone marrow stimulation. The addition of a chemokine gradient to induce cell migration or lyophilization of the scaffold to increase porosity and absorption of the cell suspension during seeding may also facilitate recellularization. Decellularization coupled with proteoglycan depletion is a promising approach for creating a biomimetic matrix that can improve clinical outcomes in cartilage repair.

## Conclusion

In this study, we established a procedure for producing a GAG-depleted collagen-based scaffold and demonstrated the potential for cells to penetrate and migrate through the matrix. Our results suggest that decellularized articular cartilage is a promising scaffold for treating focal defects, as it preserves the native collagen content and alignment and provides a supportive environment for seeded cells.
